# Farmers’ perception of impacts of bovine trypanosomosis and tsetse fly in selected districts in Baro-Akobo and Gojeb river basins, Southwestern Ethiopia

**DOI:** 10.1186/1746-6148-9-214

**Published:** 2013-10-20

**Authors:** Zewdu Seyoum, Getachew Terefe, Hagos Ashenafi

**Affiliations:** 1 Unit of Paraclinical Studies, Faculty of Veterinary Medicine, University of Gondar, P.O.Box:196, Gondar, Ethiopia; 2 Department of Pathology and Parasitology, College of Veterinary Medicine and Agriculture, Addis Ababa University, Debre Zeit, Ethiopia

**Keywords:** Baro-Akobo, Farmers’ perception, Gojeb, River basin, Trypanosomosis, Southwestern Ethiopia

## Abstract

**Background:**

Trypanosomosis, via causing anaemia, emaciation, production loss and death, is arguably the most important constraint to livestock development in Sub-Saharan countries, including Ethiopia and its impact in Baro-Akobo and Gojeb river basins (endemic areas for tsetse flies) is unknown. This study was carried out from November 2011 to April 2012 to assess farmers’ perception on the presence, impact, management and the need of intervention programs of bovine trypanosomosis and tsetse fly in selected districts located in Baro-Akobo and Gojeb river basins, Southwestern Ethiopia. A standardized questionnaire survey was employed to collect the relevant information from the farmers.

**Results:**

The result of this study showed that 94.1% of the respondents considered bovine trypanosomosis as an economically important cattle disease which accounted for 64.6% of the total annual deaths in the year 2011/2012. Estimated mean annual financial loss via mortality due to trypanosomosis was reported to be 3501 Ethiopian Birr (US$200)/household. The reported trypanosomosis suggestive signs were consistent with published reports and farmers strongly associated the occurrence of the disease with biting flies (particularly, tsetse fly). Respondents also explained that the seasonality of the disease and its vectors, i.e. May and June are peak risk months of the year. Chemotherapy was reported the major method to combating the problem, mean frequency of treatment being 5.7 times per animal per year. Because of the economic burden of the disease, farmers expressed their strong interest and support for the establishment of intervention program in their area.

**Conclusion:**

The study revealed that livestock keepers are familiar with bovine trypanosomosis and its vectors as well as its impacts. Thus, trypanosomosis and tsetse control strategies should be integrated with the local communities’ participation to minimize the impacts of the disease and its vectors in the area.

## Background

Tsetse transmitted animal trypanosomosis is an important constraint to livestock development in Africa. It occurs in around 10 million km^2^ in 37 sub-Saharan countries [1] and constitutes a major threat to the survival and productivity of domestic livestock in sub-Saharan Africa [2]. In Ethiopia, it has been described as a major impediment to the livestock development and agricultural production; contributing negatively to the overall development in general and to food self-reliance efforts of the country in particular. The annual losses to the national economy are estimated to exceed US$200 million, due to its direct and indirect impact to the agricultural and livestock production. Currently, this disease and its vector (tsetse) are excluding about 180,000-220,000 km^2^ of agriculturally suitable land of Ethiopia and also 14 million cattle, an equivalent number of shoats and nearly of a million equines are at risk of contracting the disease [3,4].

Tsetse and trypanosomosis control and eradication would benefit to promote human and livestock health, diversified agricultural systems, food production and security, and livelihood of the community and utilization of available natural resources. Several control approaches are available to eradicate trypanosomosis and its biological vector, the tsetse fly, from the area [1,5]. For the successful of these control strategies greater involvement of farmers and communities in decision making, program designing, program implementing, program evaluating and creating awareness are crucial [6-8]. Understanding of farmers’ knowledge and perceptions on the impacts of trypanosomosis and tsetse fly and their participation in developing intervention strategies are prerequisites for effective implementation [9].

Baro-Akobo and Gojeb river basins are found in the main tsetse infested tropical humid zones of Ethiopia and these are potential areas for cash crop production mainly coffee. The river basins have suitable climate for livestock production and rearing. However, livestock production is constrained by tsetse-borne trypanosomosis [10] and there have been a few reports on the prevalence of this disease [11,12]. However, the information on the impacts of trypanosomosis and its vector tsetse fly on livestock production are scanty in the area, even in Ethiopia [13]. It is, therefore; imperative to assess farmers’ knowledge and attitudes, on the presence, impact, diagnosis, treatment and control of trypanosomosis and its vectors in Baro-Akobo and Gojeb river basins. Hence, this study at a hand was conducted to assess farmers’ perception on the presence, impact, management and the need of intervention programs of bovine trypanosomosis and its vector tsetse fly in selected districts located in Baro-Akobo and Gojeb river basins, Southwestern Ethiopia.

## Methods

### Study location

The study was conducted in two selected districts located within Baro-Akobo and Gojeb river basins in Southern Nations National Peoples Regional State (SNNPR), Southwestern Ethiopia. Both river basins are found in the tsetse fly belt of Southwestern Ethiopia. Gimbo and Guraferda districts were selected from Gojeb and Baro-Akobo river basins, respectively. Baro-Akobo in the west shares international border with the eastern part of the South Sudan. Both river basins fall in the humid tropical rain forest region of Ethiopia. The annual average rainfall is among the highest in Ethiopia with a relatively dry season in January, February, March and May. Though the main rain season usually lasts from June to September; it may exceed to 8 months which is not uncommon. This trend has been, however, on gradual change. As a result, there is gradual decrease in the amount of rainfall with concomitant increase in temperature. The study areas have variable altitudes that range from 500 m to 3000 m above sea level and they are the homelands of coffee production in Ethiopia. The annual average temperature and rainfall ranges from 15.1°C to 40°C and 1200 mm to 2000 mm, respectively.

### Study design

A standardized questionnaire survey was employed to generate information on the knowledge and attitude of farmers on the presence, impact and management of bovine trypanosomosis in the study areas.

### Sampling strategy

A multistage random sampling procedure was applied as per Mahama *et al.*[14] to select districts, peasant associations and households/farmers in the study districts. The sample size of participants was determined using the formula (n = 0.25/SE^2^) given by Arsham [15] at the standard error (SE) of 0.0545 with 95% confidence interval. Animal health/production extension workers and, village community leaders and elders were involved in the identification of key informants and households that keep livestock within the area. Field assistants/enumerators with knowledge of animal health/production, familiarity with the study areas and ability to speak the local languages, were hired in order to support as well as carry out the interviews. Consequently, a semi-structured questionnaire was administered to a total of 84 randomly selected farmers/livestock keepers (42 from each district) in order to assess their knowledge on constraints of cattle production mainly on bovine trypanosomosis and its vectors. Before the interview, the objective of the research was explained to each participant and full consent of the interviewee was obtained. Identities of the livestock keepers interviewed were kept confidential to facilitate open and accurate responses. Each interview was restricted to 40–45 minutes. Initially, the semi-structured questionnaire was pre-tested to sort out the relevance of the tool. The questionnaire focused mainly on farmers’ perception on the presence, impact and management of trypanosomosis in cattle, and their desire for establishment of intervention programs against the disease. Then, major information obtained from individual interviewee was later supplemented by discussion with key informants (animal health personnel and agricultural extension works, community elders etc.).

### Statistical analysis

Microsoft Excel spread sheet program was used to manage the raw data. Both SPSS 16 [16] and STATA 11 STATA-11 [17]) statistical analysis tools were used to analyze and interpret the data. Descriptive statistics (frequency, percentage, mean, and chi-square test) were used to analyze the qualitative data. T-test was also used to compare mean annual cost of treating trypanosomosis and other diseases per house hold. Poisson regression analysis was employed to compare the mortality rates of cattle among the study districts and PA’s where cattle number of the house hold was used as exposed population. Mortality rates were calculated by adding all deaths of cattle that occurred in the house holds due to trypanosomosis during the last 12 months (only animals which showed the common signs of trypanosomosis before their death were taken into account to quantify mortalities due to trypanosomosis). Linear regression was used to compare the cost of trypanocidal drugs in relation to expenses for treating all animal diseases in the same year. Throughout the analysis, *p-*value < 0.05 was considered to have statistically significant difference.

### Ethical considerations

The permission to carry out this study was granted by Addis Ababa University (Addis Ababa, Ethiopia) and the Ethical and the Higher Degrees Committees of University of Gondar, Faculty of Veterinary Medicine (Gondar, Ethiopia), approved with a reference number: Ref. No.: RCS229/2011 how to use humans for interview and discussion purpose and all protocols or procedures in this study. We used those ethical approaches, which have been documented by Rollin [18] as a guide line. The objectives of this study were well explained to all participating farmers who all expressed their consent to participate in the interview and discussion. Questionnaire responses were given on a voluntary basis to all participants and respondents were permitted to withdraw their consent up to three days before data submission.

## Results

### Constraints associated with cattle production

Respondents reported different constraints that hinder the success of cattle production. Diseases, shortage of feed, improper grazing land management, lack of institutional support, inadequate veterinary services and market problem were most frequently reported constraints of cattle production in the study districts. Diseases resulting in high mortality and morbidity were perceived by 96.4% of livestock owners’ or respondents as the most important constraints associated with cattle production. As indicated in Table 1, trypanosomosis (95.2%), fly strike/nuisance (66.7%), ectoparasite (tick) infestation (52.4%), pasteurellosis (42.9%), blackleg (35.7%) and internal parasites (33.3%) were most frequently reported diseases in that order.

**Table 1:** The reported common types of cattle diseases in the study districts or river basins (n, %)

**Diseases**	**Gimbo**		**Guraferda**		**Total**	
	**n**	**Proportion (%)**	**n**	**Proportion (%)**	**n**	**Proportion (%)**
Trypanosomosis	39	92.9	41	97.6	80	95.2
Fly strike/nuisance	25	59.5	31	73.8	56	66.7
Ectoparasite	25	59.5	19	45.2	44	52.4
Pasteurellosis	16	38.1	20	47.6	36	42.9
Black-leg	20	47.6	10	23.8	30	35.7
Internal parasite	16	38.1	12	28.6	28	33.3
Mastitis	19	45.2	5	11.9	24	28.6
Sudden death	13	31	10	23.8	23	27.4
Leech problem	9	21.4	12	28.6	21	25
Lameness	11	26.2	4	9.5	15	17.9
Anthrax	8	19.1	6	14.3	14	16.7
Lumpy skin disease	9	21.4	-	-	9	10.7
Foot and mouth disease	8	19.1	-	-	8	9.5

(n- represents number of respondents).

### Farmers’ perception on the presence and impact of tsetse and trypanosomosis

Most livestock keepers 95.2% (92.9% in Gimbo and 97.6% in Guraferda) reported that they are familiar with bovine trypanosomosis (locally called “*Gendi* or *Golebo*”). Among these, 94.1% of the respondents listed bovine trypanosomosis as the prime economically important cattle disease in the river basins/districts. According to the respondents, among the 65 cattle deaths reported (Table 2), trypanosomosis accounted for 64.6% of the total annual deaths in the last 12 months of the year (2011/2012) and the mortality rate of cattle was 6.4%. The estimated mean financial loss due to cattle mortality as a result of bovine trypanosomosis was about 3502 ETB (US$ 200; “according to NBE 1 US$ is equivalent to 17 ETB”) per household in the study districts. However, key informants and greater number of the respondents (77.8%) said that the status/trends of trypanosomosis and its biological vector density now a day’s is decreasing gradually. In contrast to this, 16.1% of respondents said that the risk of fly and the disease has not changed while 6.2% of them reported the challenges increased time to time, and the difference between these two groups of respondents was statistically significant (*P* < 0.05).

**Table 2:** Mean mortality rates and estimated financial loss due to bovine trypanosomosis in the year (2011/2012)

**District**	**Total deaths**	**Deaths due to tryps**	**Cattle owned by respondents**	**Mortality rates due to tryps (%)**	**Loss (in birr) due to tryps**
Gimbo	29	21	388	5.4	3090.0
Guraferda	36	21	269	7.8	3913.3
**Total**	**65**	**42**	**657**	**6.4**	**3501.7**

Ninety-five percent of respondents in Guraferda district and eighty six percent in Gimbo district were aware of the causal association between flies (biting flies) and bovine trypanosomosis. Some respondents also associated trypanosomosis with tick bite, watering animals at river, river side grass grazing and drinking stagnant water. 44.6% of respondents also reported that wild animals are important sources of trypanosomosis etiology.

**Figure 1:**
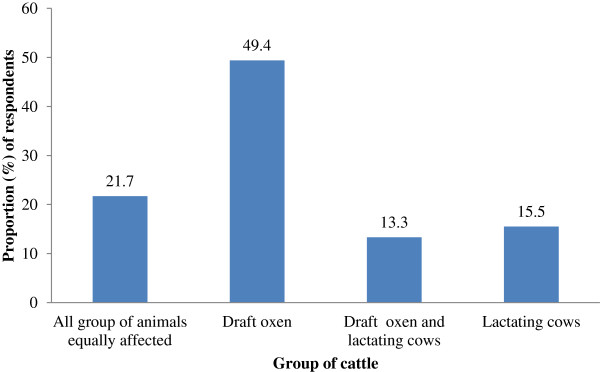
Group of cattle most severely affected by trypanosomosis as perceived by owners.

49.4% of livestock keepers have stated that bovine trypanosomosis is most severe in draft oxen (Figure 1) than in other groups of cattle whereas 21.7% asserted that the disease is equally severe in all group of animals. On the other hand, 13.3% and 15.5% of the respondent farmers noted that more trypanosomosis problem has been encountered in draft oxen and lactating cows, and lactating cows alone, respectively. Draft power loss due to sickness of oxen, treatment cost, production losses (milk and growth reduction), interference with agricultural activities, induced mortalities and replacement cost were considered as important impact of bovine trypanosomosis for 68.3%, 53.7%, 48.8%, 45.15%, 35.4% and 25.6% of the respondents, respectively.

### Farmers’ perception of signs of bovine trypanosomosis

Livestock owners’ noticed that different clinical signs of trypanosomosis that could be easily identified through visual observation. Though, the level of precision depends on the experience of the livestock keepers, among the observed signs of bovine trypanosomosis: ruffled hair, progressive emaciation, physical weakness, anorexia, and reduced production and performance were the most frequently reported clinical signs of trypanosomosis by livestock keepers (Table 3). In addition to this, respondents emphasized that in cattle suspected of trypanosomosis, noticeable reductions could be observed on milk production, body condition, working ability of oxen, growth rate, and price of the animal in addition to increased mortality rate in untreated cases.

**Table 3:** Frequently observed clinical signs of trypanosomosis according to respondents’ perception

**Clinical signs**	**Gimbo (N = 42)**		**Guraferda (N = 41)**		**Total (N = 83)**	
	**n**	**%**	**n**	**%**	**n**	**%**
Ruffled hair	38	90.5	28	68.3	66	79.5
Progressive emaciation	36	85.7	22	53.7	58	69.9
Physical weakness	36	85.7	22	53.7	58	69.9
Anorexia	27	64.3	24	58.5	51	61.5
Reduced production/performance	29	69.1	13	31.7	42	50.6
Eating soil	14	33.3	22	53.7	36	43.4
Diarrhoea	15	35.7	16	39.0	31	37.4
Constipation	10	23.8	13	31.7	23	27.7
Lacrimation of eyes	11	26.2	11	26.8	22	26.5
Death	7	16.7	7	17.1	14	16.9
Coughing	3	7.1	8	19.5	11	13.3
Abortion	7	16.7	3	7.3	10	12.1
Swelling of lymph node	6	14.3	4	9.8	10	12.1
Loss of hair	6	14.3	2	4.9	8	9.6
Salivation	4	9.5	3	7.3	7	8.4
Others	9	21.4	8	19.5	17	20.5

### Perception on seasonality of trypanosomosis and tsetse fly challenges

**Figure 2:**
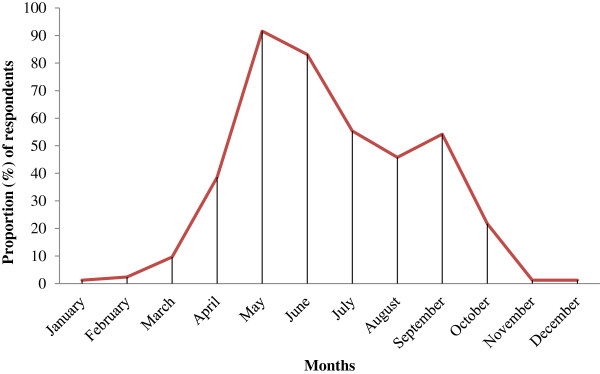
Monthly variation in the risk of trypanosomosis and tsetse fly as perceived by farmers.

Interviewees and key informants have declared that trypanosomosis and tsetse fly challenges have wide seasonal variations (Figure 2). About 64.2% of the respondents explained that trypanosomosis and its vectors challenge reach peak level at the beginning of long rainy season (end of April-June) in terms of profound morbidity and mortality effect of the disease, fly density and fly nuisance effect. In this aspect, there was no difference (*P* > 0.05) among districts. On the other hand, majority of the respondents ascertained that the challenges of trypanosomosis and its vectors become mild in the dry season, particularly, in November, December, January and February.

### Perception on the management of trypanosomosis and its cost

Overall, trypanocides 96.3% (79/82) followed by anthelmintics 67.1% (55/82), antibiotics 47% (39/82) and acaricide 35.4% (29/82) (to control ectoparasites) have been most commonly used drugs against cattle illness and ectoparasite infestation in the study districts. Use of trypanocides and ecto-pour (mainly in Guraferda district) were the only ways of managing/combating bovine trypanosomosis in the study areas at the time of this study. However, livestock keepers complain against ecto-pour/pour-on, a pour-on permethrin-piperonyl butoxide insecticide because it causes hair loss and skin damage on the back of cattle. Among those responded to have used trypanocides, most indicated Diaminazine aceturate and Isometamidium chloride (Veridium) as the most common drugs used in the study districts. These drugs have been mainly sourced from public veterinary clinics, private veterinary drug shops/stores and local markets. In this aspect, veterinary public health clinic is the main drug source in Guraferda district for 92.7% and in Gimbo district for 62% respondents; which was significantly different (*P* < 0.05). In addition; according to the livestock keepers report, trypanocidal drugs are commonly administered by animal health personnel (animal health assistants and community animal health workers) while significant number of cases were reported to be treated by animal owners or their family members.

The average number of trypanocidal treatments per year/cattle as indicated by the respondents was 5.7 times (range: 1–12). Mean number of treatments per individual animal per year was 7.4 times in Guraferda and 4.6 times in Gimbo district and the difference between districts was statistically significant (*P* < 0.05). According to the respondents, the mean (±σ) cost of trypanocidal treatment per single dose was 10 ± 2 ETB. It was higher in Gimbo (11 ± 2) than Guraferda (9 ± 2) and the difference is significant (*P* < 0.05). Therefore, on an average livestock keepers spent about 55 ± 14ETB (3US$) for single large animal for trypanosomosis per annum. Moreover, the average (±σ) amount of money expenditure per household per year to purchase trypanocidal drugs in the study areas for the treatment of bovine trypanosomosis was 225 ± 121 ETB (13 ± 7 US$) (Table 4). Over all costs of treating trypanosomosis was significantly (*P* < 0.05) higher than all costs of treating other diseases in both districts. Costs for treating other diseases in the study districts were mainly due to the purchase of antibiotics, acaricide and anthelmintics.

**Table 4:** Mean annual cost of treating trypanosomosis and other diseases per house hold per year

**Districts**	**Treatment cost of trypanosomosis (ETB)**	**Treatment cost of other diseases (ETB)**	**All treatment costs (ETB)**
Gimbo	207.9	63.8	266.34
Guraferda	241.9	35.9	280.7
**Mean total cost**	**224.5**	**49.9**	**273.2**

(t = 3.9, df = 77, *P* < 0.05).

### Willingness of livestock owners for establishment of intervention program

According to the information obtained, there was no launched governmental or non-governmental tsetse and trypanosomosis intervention activity in the study areas. Almost all (96.3%) of the respondents, had positive attitude for the establishment of trypanosomosis and tsetse fly intervention program in their surroundings. 87.7%, 35.4% and 30.5% of the livestock keepers expect fly and disease eradication, treatment services and drug supply benefits from the coming project, respectively. Moreover, most (92.7%) interviewees and key informants have stated their willingness to contribute their support (by money, labor and/or protection) if intervention program will be established in their surroundings.

## Discussion

In order to improve the welfare and security of rural communities in Africa, particularly Ethiopia, rapid method for assessing risk and diagnosing urgent problems are needed for the control of both human and animal diseases. Therefore, this survey was conducted with the objective of seeking information about constraints of cattle production, diseases of cattle particularly the impact of bovine trypanosomosis and its management in selected districts in Baro-Akobo and Gojeb river basins. The results of the survey indicated that diseases resulting in high mortality and morbidity were highly prevalent in both districts and perceived as the most important constraints associated to cattle production. Of the reported diseases, trypanosomosis has been perceived as the number one obstacle to cattle production in the study areas. In agreement with the report of Rowlands *et al.*[19], the ranking of trypanosomosis as the first most important constraint to cattle production is not surprising given that both study districts were tsetse infested and that the disease causes observable losses in many aspects of production. Similarly, Stein *et al*. [10] observed that most respondents from tsetse infested parts of Ethiopia considered trypanosomosis as the main animal health impediment in their areas. In Afework *et al.*[20] and Tewelde *et al.*[21] studies, farmers strongly recognized trypanosomosis as the primary problem for livestock productivity and agricultural development in the northwestern and western parts of Ethiopia, respectively. This is also in agreement with reports from other tsetse infested parts of the country and elsewhere in Africa [22-27]. Survey conducted in tsetse and trypanosomosis control project areas in Nigeria showed that tsetse and trypanosomosis are of much concern to farmers and represents as major obstacle to livestock production and development of allied industries [2].

In the present survey, trypanosomosis accounted for significant annualized cattle deaths. This is in agreement with the reports of Muturi [28] and Tesfaye *et al*. [27] who recorded 6.5% and 4.4% mortality rate of cattle in southern rift valley and Northwest Ethiopia. In Africa, more than 3–7 million cattle die due to bovine trypanosomosis (about 6% mortality rate per year) from more than 50 million cattle population at risk in the tsetse-infested areas [29,30]. The estimated mean financial loss via cattle mortality as a result of bovine trypanosomosis in this current survey is significantly higher than other reports in the country and elsewhere in Africa [27].

Significantly higher numbers of respondents in the study districts are aware of the causal association between biting flies and bovine trypanosomosis but not considered as the etiological factor. This coupled with their knowledge on the signs of the disease and treatment suggests that the farmers have comparable understanding of the problem as reported earlier [9,25,31] in other African countries.

Though, the level of precision depends on the experience of the livestock keepers, most farmers could determine clinical signs suggestive of bovine trypanosomosis that are commonly described for the disease [5,32]. Similarly, studies conducted in tsetse-infested areas of Ethiopia [10,13,26,27], Kenya [9,25,33] and other west African countries [31] have revealed that most of the interviewed livestock farmers were able to mention the common symptoms that are used as diagnostic tool for trypanosomosis suspected cases.

According to respondents in the present survey, bovine trypanosomosis is most severe in draft oxen followed by lactating cows. This might be attributed to stress associated with work overload, lactation and pregnancy. As Rowlands *et al*. [34], Okech *et al*. [35], Swallow [30] and, Taylor and Authié [36], animals with work overload, lactation and pregnancy are highly susceptible to the diseases. In addition to this, respondents have emphasized that in cattle suspected of trypanosomosis, noticeable reductions could be observed on milk production, body condition, working ability of oxen, growth rate and price of the animal and increased mortality in untreated cases. This is in accordance with Swallow [30], Shaw [37] and Radostits *et al.*[32] who pointed out the impact of trypanosomosis.

Ability of livestock keepers to determine seasonal variations and peak level of trypanosomosis and its vectors as recorded in the present study is in line with the reports of Chernet *et al*. [38], Shimelis *et al*. [26] and Tesfaye *et al*. [27] from Ethiopia, Catley *et al*. [33] from Kenya and Grace *et al*. [31] from west Africa. Wet and warm months of the year (such as May and June) are favorable periods for vectors and trypanosomes growth and multiplication and are observed as peak months of the disease. This could be attributed to the favorable temperature and moisture for tsetse fly puparium development. Most livestock keepers have strongly associated the disease with bites of tsetse flies followed by *Stomoxys* and *Tabanus* species of flies. Similarly, in Catley *et al*. [33], Machila *et al*. [9], Ohaga *et al*. [25] and Stein *et al*. [10] studies, farmers have suggested tsetse fly as prime transmitter of bovine trypanosomosis but did not consider being the sole etiological factor for the disease. In addition to this, livestock keepers have also identified river side, forest, bushy grass land and grazing area as the most risky places for fly and trypanosomosis exposure. This is in line with the scientific description about the biology and ecology of the flies [39,40].

To treat sick animals suspected of trypanosomosis, livestock keepers have relied upon the use of modern veterinary drugs, mainly Diaminazine aceturate and Isometamidium chloride (Veridium) whereas ecto-pour, which was disliked by most farmers due to its side effect on the skin, was reported to be employed against flies in one of study districts (Guraferda). However, the high frequency of trypanocidal application coupled with the report of self preparation and injection of the drugs by significant number of farmers indicates that there is high risk of development of drug resistance in the areas. According to Geerts and Holmes [41], repeated use of chemicals as pesticides or chemotherapeutic agents inevitably leads to the development of resistance in the target organism. Van den Bossche *et al.*[42], Holmes *et al.*[43] and Radostits *et al.*[32] also stressed that prolonged or frequent use of trypanocidal drugs in high challenge areas results in high selection pressure for resistance or causes development of drug resistant or drug fastness. The frequency of treatment reported in the present study is higher than earlier reports from other parts of Ethiopia [20,21,26-28]. Therefore, this observation deserves further study on the efficacy of the common trypanocidal drugs that are being used.

In general, according to Uilenberg [44] and Maudlin *et al.*[5], the number of treatments over a year reflects the magnitude of trypanosome and tsetse fly challenge and drug resistance presence in the area. In agreement with this, the mean financial expenditures to purchase trypanocidal drugs per household per year in the study areas were higher than all costs of treating other diseases in both districts. This is in accordance with the work of Tesfaye *et al.*[27] that had been conducted in highly tsetse infested areas of northwest Ethiopia. The absence of organized governmental or non-governmental intervention program related to trypanosomosis and tsetse fly in the study areas, the expressed need for the establishment of intervention scheme and farmers’ willingness to support in many aspects also suggests the severity of the problem and the need for urgent action. This is in line with previous findings in East and West Africa [45-49].

## Conclusion

The study conducted on the livestock keepers’ perceptions about tsetse fly and trypanosomosis in Baro-Akobo and Gojeb river basins, provided important information on the disease situation and farmers’ perception on impact of the disease. In this study, livestock owners are familiar with bovine trypanosomosis and strongly acknowledged that it is the main sanitary constraint to livestock development in Baro-Akobo and Gojeb river basins. Livestock owners also identified the vector, tsetse fly and associated it with the disease trypanosomosis. They also had good knowledge on the suggestive signs of trypanosomosis and its impact on the agricultural activity and on the livelihood and well-being of cattle as well as on the owners themselves. Moreover; livestock keepers in the present survey expressed their strong desire for the establishment of intervention program in their areas. In addition, they expressed their willingness to support any intervention project to be implemented. In general; farmers are fully perceived and well aware of bovine trypanosomosis and its vector, impact, seasonality and intervention options. Hence, it is advisable to incorporate farmers’ knowledge in designing and implementing of the intervention program in the areas.

## Competing interests

The authors declare that they have no competing interests.

## Authors’ contributions

ZS: Developed the project, wrote the protocol, collected the data during field work, analyzed the data and interpreted the result, drafted the manuscript and final proof reading of the manuscript before was submitted to the journal. GT: Developed the project, Project leader, gave support during field work, read, reviewed and corrected the manuscript on technical and language part. HA: Developed the project, Assistant project leader, read, edited and gave technical support on the manuscript before was submitted to the journal. All authors read and approved the final manuscript.
